# Alternative Approach to the Management of Postoperative Pain after Pediatric Surgical Procedures

**DOI:** 10.5005/jp-journals-10005-1249

**Published:** 2014-08-29

**Authors:** Marco Paschoal, Juliana Souza, Lourdes Santos-Pinto, Cyneu Pansani

**Affiliations:** PhD Student, Department of Pediatric Dentistry, Araraquara Dental School Univ Estadual Paulista-UNESP, São Paulo, Brazil; PhD Student, Department of Pediatric Dentistry, Araraquara Dental School Univ Estadual Paulista-UNESP, São Paulo, Brazil; Professor, Department of Pediatric Dentistry, Araraquara Dental School Univ Estadual Paulista-UNESP, São Paulo, Brazil; Professor, Department of Pediatric Dentistry, Araraquara Dental School Univ Estadual Paulista-UNESP, São Paulo, Brazil

**Keywords:** Low-level laser therapy, Pediatric dentistry, Post-operative pain

## Abstract

**Aim:** This paper reports two clinical cases in which the application of low-level laser therapy (LLLT) enhanced the postoperative symptoms after pediatric surgical procedures.

**Background:** The uses of novel technologies allow more comfort to the patients and ensure a rapid procedure, and LLLT application has shown a positive effect in the prevention of discomfort after invasive procedures.

**Case description:** Low-level laser therapy protocol was applied after surgical removal of supernumerary tooth and frenectomy resulting in less swallow and pain with no need of medication intake.

**Conclusion:** The laser application was well accepted by both children and parents and showed a clinical efficiency in the follow-up examinations beyond the satisfactory quality of wound healing.

**Clinical significance:** The LLLT approach is an excellent adjuvant therapy resource for delivery an optimal postoperative after surgical procedures in children.

**How to cite this article:** Paschoal M, Souza J, Santos-Pinto L, Pansani C. Alternative Approach to the Management of Postoperative Pain after Pediatric Surgical Procedures. Int J Clin Pediatr Dent 2014;7(2):125-129.

## INTRODUCTION

The management of pediatric patient’s behavior in the dental office is a challenge for dentists when surgical procedures are needed.^1,2^

The pain relieve prescription has been a great point of discussion among professionals. It is largely known the positive effects related to efficacy and safety of nonsteroidal anti-inflammatory drugs (NSAIDs) and analgesics in general dental practice.^3^ However, potential adverse effects of these medications included peptic ulcer disease, gastrointestinal bleeding, impaired renal function and inhibition of platelet function. So, when patients are focused, especially children, there is a need to depend on another analgesic tool with minimal side effects.^4^

The low-level laser therapy (LLLT) application is thought to reduce pain, accelerate wound healing and reduce the inflammatory process, with no side effects.^5,6^ Moreover, LLLT seems to present positive effects in biomodulation, analgesic effects and with stimulating action in tissue repair and wound healing. The exact biological mechanisms induced by LLLT, however, are not fully elucidated.^5,7^ Altered cellular functions, as ATP, protein and prostaglandin synthesis, phagocytosis, neurotransmitter release, cell growth and differentiation as well membrane potentials and binding affinities have been discussed to be responsible for the low energy laser effects.^8^

In relation to reduction of pain, the most acceptable theory is that effective pain reduction can be achieved via increase in β-endorphins, blocked depolarization of C fiber afferent nerves, increased nitric oxide production, increased nerve cell action potential, axonal sprouting and nerve cell regeneration, decreased bradykinin levels, increased release of acetylcholine or ion channel normalization.^9,10^

Many clinical studies and case reports investigated the use of LLLT applications. Positive laser effect was used for the prevention of pain, swelling or trismus after removal of third molars and periodontal surgery procedures as well as for reducing orthodontic postadjustment pain.^11,12^ While some studies reported beneficial effects of LLLT, others showed no or only negligible clinically relevant influence of the application.^13^ Thus, the literature does not support reliable clinical decisions, becomes controversial in the actual role of LLLT in dental practice.

Apart of the unclear outcomes, LLLT seems to be an important tool that can provide more comfortable and faster postoperative recovery for patients after several kinds of surgery, such as tooth removal.^6^

In pediatric field, offer new possibilities and alternatives to improve quality of life of patients submitted to surgical procedures aiming to provide a more comfortable postoperative symptoms, and a faster recovery is mandatory when the issue is the management of pain.^6,14^ Hence, the objective of this present paper is to report two clinical cases in which the application of LLLT enhanced the postoperative symptoms after pediatric surgical procedures.

## CASE REPORTS

### Case 1

**Figs 1A and B:**
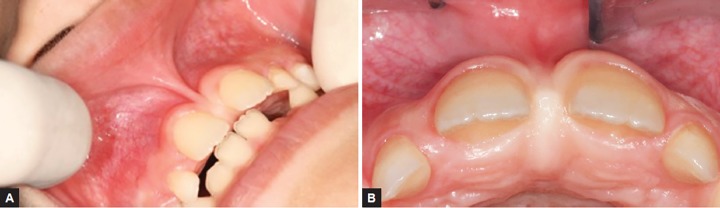
(A) Frontal view of the frenum. Notice the ischemia of the area due to membrane tension, (B) occlusal view of the incisive pappilae. Notice the ischemia of area after the tension of the frenum

A 9-year-old boy was referred to the Pediatric Clinic, Araraquara Dental School, Univ Estadual Paulista-UNESP, aiming to perform frenectomy surgery for orthodontic needs. After evaluation of the case and completion of medical and dental history, neither contraindications to perform the procedure nor systemic disease were verified. In the intraoral examination, the abnormal attachment of the upper labial frenum was verified and presented too close to the marginal gingiva resulting in local ischemia when the mucous membrane was tensioned (Figs 1A and B). The intervention was explained to the parents, and surgical procedure was performed.

The soft tissue received immediately to postoperative period, an irradiation of an gallium-aluminum-arsenide (GaAlAs) diode low lever laser (Thera Lase, DMC, São Carlos, SP, Brazil) in the visible red wavelength (685 nm) with a mean output power of 35 mW (0.1 W) in continuous mode with a spot size of 1 mm aimed to accelerate the wound healing process. The area was exposed into 4 points that were irradiated for 49 seconds with delivery energy of 4.9 J for each point with an energy fuency of 30 J/cm^2^. This protocol was performed 24 and 48 hours after the surgical procedure. To the laser application, both operator and patient wore appropriate glasses for eye protection and the laser probe was disinfected with 70% alcohol solution and wrapped with a plastic protection. Analgesic (Ibuprofen 600 mg, Glaxo SmithKline Brasil Ltd, Rio de Janeiro, RJ, Brazil) was prescribed and the patient was instructed to use it when needed. During the first postoperative days, the patient was instructed to note pain intensity using a horizontal 10 cm visual analog scale (VAS) and any possible side effects (e.g. dizziness, nausea) also time and intake medication if needed. The VAS is constituted by marks ranging from 0 (without pain) to 10 (the worst pain imaginable).^15^ Degree of pain was evaluated at the first hour and at 2, 4, 8, 12 hours after the completion of the surgery and for the next 2 days. For all the periods evaluated, the patient related neither a discomfort (0 of pain degree) nor need of medication intake.

**Fig. 2:**
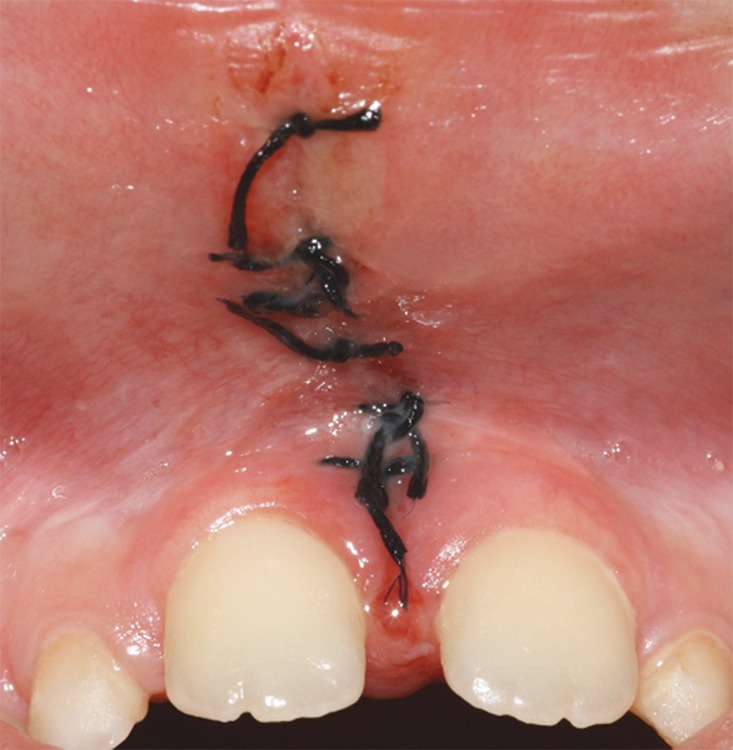
Aspect of the surgical area after 7 postoperative days

**Fig. 3:**
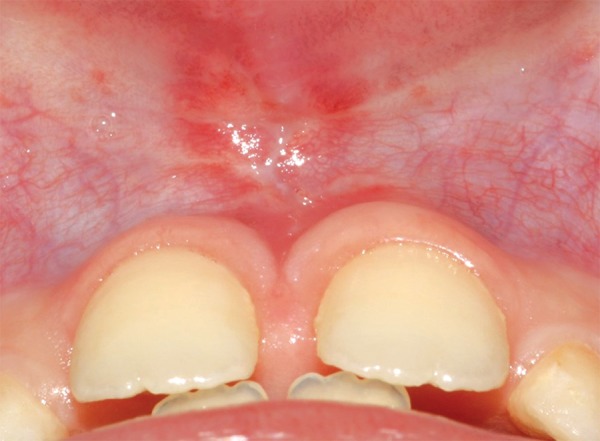
Aspect of the surgical area after 15 postoperative days

**Fig. 4:**
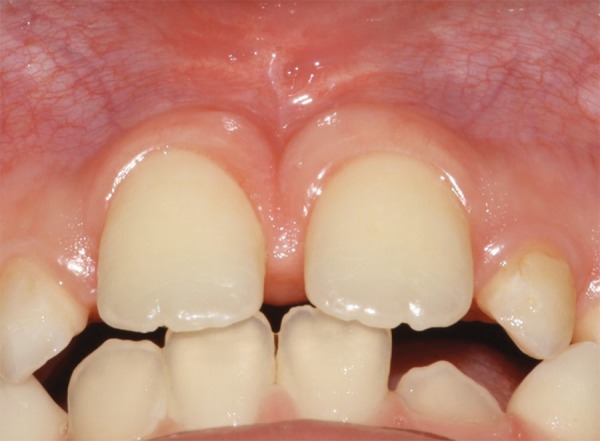
Aspect of the surgical area after 3 months follow-up

After 7 days, the patient returned for suture removal and was noted no infection or swelling (Fig. 2). Figure 3 showed the aspect of the surgical area after 15 postoperative days. A 3 months follow-up showed the quality of wound healing and tissue repair (Fig. 4).

### Case 2

**Fig. 5:**
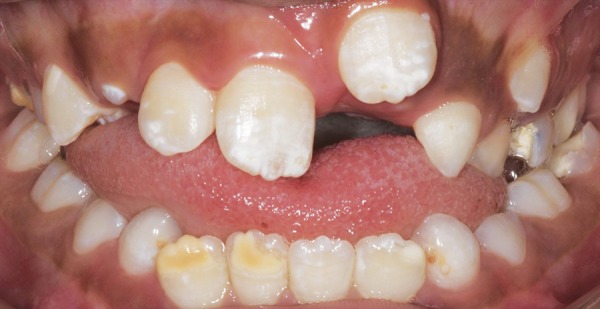
Intraoral view of the unerupted left permanent upper lateral incisor

**Fig. 6:**
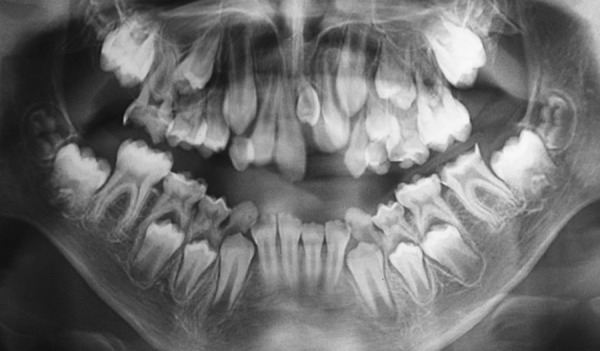
Panoramic radiograph attesting the presence of the supernumerary tooth in the anterior region of maxilla

A 10-year-old boy presented to the Pediatric Clinic, Araraquara Dental School, Univ Estadual Paulista-UNESP with the main complaint of misaligned teeth. An intraoral examination revealed the absence of the left permanent upper lateral incisor (Fig. 5). The patient was submitted to panoramic radiograph that confirmed the hypothesis of the presence of the supernumerary tooth located near of the maxillary left central incisor (Fig. 6). Periapical radiograph was taken to confirm the location of the supernumerary tooth. After mother’s speech, it was verified that the patient presented healthy with positive dental and medical behavior prior and during the routine appointments. The need of the surgical procedure also the remotion of the tooth was explained to both the patient and mother.

Immediately after the surgical procedure, the region of the extracted supernumerary tooth received an irradiation of the GaAlAs diode low-level laser (Thera Lase, DMC, São Carlos, SP, Brazil) in the invisible wavelength (830 nm) in two different points aiming to increase bone formation. Each point was illuminated for 17 seconds and was delivered 3.4 J of energy with the fluence of 60 J/cm^2^, and the mean output was adjusted to 100 mW (0.1 W). The region that received the sutures was also irradiated into four different points. To these applications, the laser device was adjusted in the red visible light wavelength and the parameters utilized were similar to the anterior case described above, since the reason for the irradiation was increase the quality repair of the soft tissue. This protocol was applied after 24 and 48 hours after the surgical procedure.

The pain recorded in the VAS of the patient showed that apart from the period of 12 hours in which the patient marked a number between 2 and 3 thus demonstrating an annoying sensation. The other periods evaluated it was demonstrated a mild to none pain sensation (between 1 and 0). Furthermore, the patient reported no need to take any medication.

**Fig. 7:**
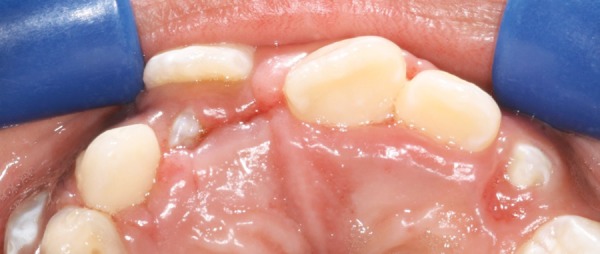
Aspect of the intervention area after 7 postoperative days

The patient was recalled after 7 days to remove the sutures and the aspect of the area of intervention presented no signs of hemorrhage and swelling. The soft tissue presented a high level of healing accompanied of satisfaction of the mother with the procedure (Fig. 7).

**Fig. 8:**
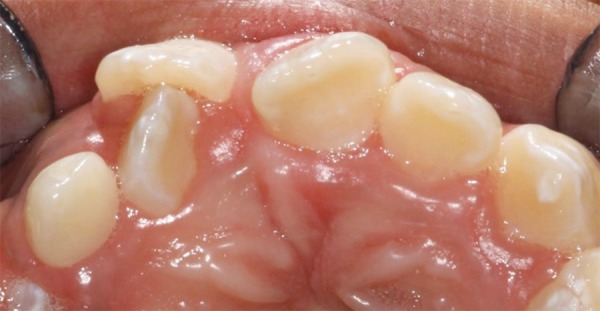
Aspect of the area after 3 months follow-up. Notice the presence of the left upper lateral incisor and left permanent upper lateral incisor

A follow-up of 3 months was performed and revealed the health aspect of the gingival contour and the presence of left permanent upper lateral incisor (Fig. 8).

## DISCUSSION

The most valuable treatment goal in dental practice, especially in pediatric field, is to afford the patient a pain-free treatment.^16^ Furthermore, offer a treatment with mild or no painful sensation is effective to the success of the therapy.

The utilization of laser as a nonsurgical medical approach for assisting the normal processes of healing has increased over the last few years. Laser treatment is an innovative method and has been shown to produce several different effects, including pain relief, wound healing, bone formation and nerve regeneration. It has potential biostimulating effects when applied to oral tissues, e.g. increase the wound healing quality, improve epithelization after surgical procedures, minimizing edema and preventing oral mucositis.^17-19^

In the presented case reports, the application of low laser therapy after surgical procedures achieved an excellent outcome since the patients did not intake any medication for pain relief and optimal quality of wound healing in a short period of time.

There are many clinical studies concerning laser therapy as an adjunct tool to enhance the postsurgical recovery period. Positive laser effect was used for the prevention of pain, swelling or trismus after removal of third molars and periodontal surgery procedures as well as for reducing orthodontic postadjustment pain.^11,12^

The protocol utilized in this present approach was based in a previous study and followed the manufacturer’s instructions.^20^ However, the LLLT protocol applied here was fully described; many investigations do not show this information clearly in the body of text content. Furthermore, the irradiation parameters (e.g. fuency, mode of operation, laser wavelength, output power) which are the basis success of the LLLT were utilized in different ways becoming comparisons among studies a hard task.

In pediatric dentistry, the recovery period after surgical procedures must be as comfortable as possible. In this way, a surgical wound healing that is free-infection and with reduced presence of inflammation and pain is extremely necessary.^6,21,22^ Furthermore, the presence of laser device reduces the perception of fear in the patients, thus, encourage a positive attitude toward the dental treatment.^15^ The results exhibited in this study is in agreement of a previous approach that suggested the laser treatment results in minimum or nether postoperative swelling nor pain with less discomfort.^23^

## CONCLUSION

The LLLT approach represents an excellent adjuvant therapy resource for delivery an optimal postoperative after surgical procedures in children.^24^ Additionally, this method is simple, low-cost and is a safe therapy with no side effects and, at same time, reduces the use of anti-inflammatory drugs.

## CLINICAL SIGNIFICANCE

The LLLT approach is an excellent adjuvant therapy resource for delivery an optimal postoperative after surgical procedures in children.
